# Migration of the Anterior Spinal Rod to the Right Thigh, a Rare Complication of Anterior Spinal Instrumentations: A Case Report and a Literature Review

**DOI:** 10.1155/2015/532412

**Published:** 2015-11-03

**Authors:** Camino Willhuber Gaston, Taype Zamboni Danilo, Carabelli Guido, Barla Jorge, Sancineto Carlos

**Affiliations:** Institute of Orthopedics “Carlos E. Ottolenghi,” Italian Hospital of Buenos Aires, Juan D. Peron 4190, C1181ACH Buenos Aires, Argentina

## Abstract

Posterior and anterior fusion procedures with instrumentation are well-known surgical treatments for scoliosis. Rod migration has been described as unusual complication in anterior spinal instrumentations; migration beyond pelvis is a rare complication. A 32-year-old female presented to the consultant with right thigh pain, rod migration was diagnosed, rod extraction by minimal approach was performed, and spinal instrumentation after nonunion diagnosis was underwent. A rod migration case to the right thigh is presented; this uncommon complication of spinal instrumentation should be ruled out as unusual cause of sudden pain without any other suspicions, and long-term follow-up is important to prevent and diagnose this problem.

## 1. Introduction

Surgical treatment for progressive lumbar scoliosis can be performed by anterior or posterior fusion procedures. Multiple complications related to spinal instrumentation have been described as wound infection, nonunion, and neurological compromise [1, 2]. Rod migration after spinal instrumentation has been reported previously in few cases [3, 4]. Since the introduction of anterior scoliosis fusion, with important contribution from Kaneda et al. [5], Chan [6], and Moe et al. [7], advantages of this procedure such as fewer spinal fusion segments, avoidance of crankshaft phenomenon, and less blood loss have been described. On the other hand, complications related to this technique include vascular, neurological, ureteral, and gastrointestinal injuries as well as implant failure and nonunion. Rod migration beyond the pelvis is particularly a rare complication.

We report a rod migration from an anterior lumbar spinal fusion through the psoas muscle to the right thigh; surgical treatment and definitive fusion are presented as well.

## 2. Case Report

A 32-year-old female presented to our outpatient department with complaint of acute right thigh pain. Physical examination at presentation showed pain that increased with active movement and palpation at the anterior and medial aspect of the right thigh.

The patients referred to a spinal surgery 12 years ago for scoliosis treated in another institution as the only surgical background.

**Figure 1:**
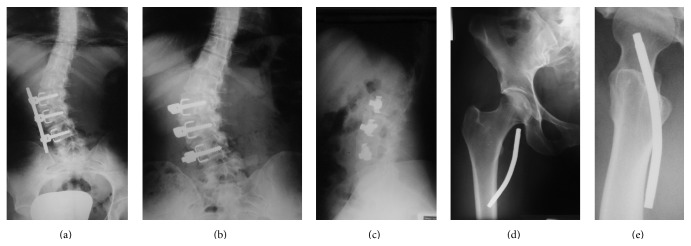
(a) Previous anterior view of lumbar spine. (b-c) Anterior and lateral views of lumbar spine, a three-level instrumentation without the anterior rod. (c-d) Anterior and lateral right hip views showing the rod migration.

**Figure 2:**
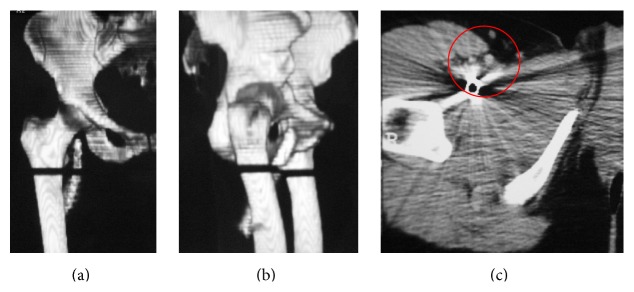
(a-b) CT-scan reconstruction. (c) Rod closeness to the femoral vessels.

After ultrasonography and radiography, a foreign body was observed: Isola instrumentation from L2–L4 with rod material from lumbar spine which had migrated and produced pain (Figure 1). A computerized angiotomography showed no rod contact with femoral vessels (Figure 2).

**Figure 3:**
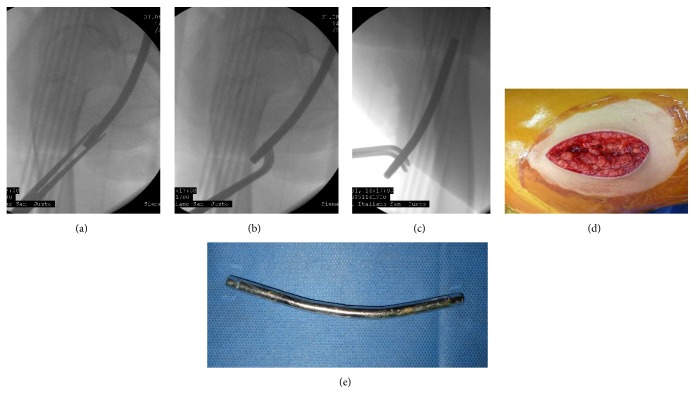
(a–c) Radioscopic rod pulled out. (d) Minimal anterior thigh approach. (e) Rod material removed.

At August 31, 2012, the surgery was performed, through a minimal anterior-medial approach, and the rod was identified and removed with radioscopic guidance; no complications were observed during the surgical procedure (Figure 3).

One year after surgery, the patient consulted for chronic lumbar pain; after clinical examination, X-rays, and MRI studies, nonunion was diagnosed and the lumbar curve (T12 to L5) showed 45 degrees with Modic 1 changes at L5–S1.

**Figure 4:**
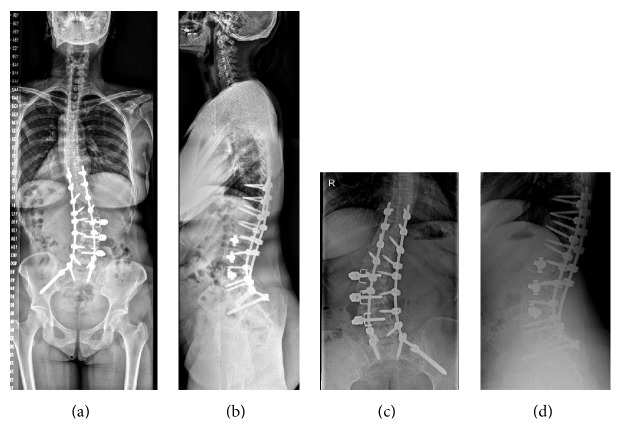
(a-b) Anterior and lateral spinal views with definitive instrumentation T-10 to iliac bone. (c-d) Lumbar X-ray views.

Spinal instrumentation was proposed and a T10 to iliac arthrodesis with L4–L5 and L5–S1 combined anterior transforaminal lumbar interbody fusion was performed (Figure 4).

## 3. Discussion

Surgical treatment for scoliosis can be performed by anterior or posterior fusion procedures. For anterior rod instrumentation, fewer spinal fusion segments are usually required; in addition, avoidance of crankshaft phenomenon and less blood loss have also been reported as advantages in comparison to posterior rod instrumentation.

Rod migration in anterior spinal instrumentation is an uncommon complication but can result in high morbidity and fatal outcomes. Instrumentation failure could be explained because of motion in the setting of nonunion in fusion attempts [3, 4, 8]. Migration through retroperitoneal lumbar segment to the pelvis and thigh following an iliopsoas pathway close to iliac and femoral vessels has been reported previously [4, 9].

Nearly half of all migrations are asymptomatic [10] but sometimes can cause severe complications, such as those reported by Al-Binali et al. [11] who described a gastrointestinal bleeding because of rod migration to the internal iliac artery as well as the rectal wall. Banit et al. [12] reported a sacral fixation rod migration to the acetabulum in which a reoperation was required.

Early stages of rod migrations are usually asymptomatic [4, 9], the systematic follow-up with clinical and radiological examination is then important to prevent or minimize such complication [13].

In conclusion, an uncommon complication of anterior arthrodesis was presented, and clinical suspicion and surgical treatment were described.
