# Corrigendum to “Migration of the Anterior Spinal Rod to the Right Thigh, a Rare Complication of Anterior Spinal Instrumentations: A Case Report and a Literature Review”

**DOI:** 10.1155/2016/7287190

**Published:** 2016-10-11

**Authors:** Gaston Camino Willhuber, Danilo Taype Zamboni, Guido Carabelli, Jorge Barla, Carlos Sancineto

**Affiliations:** Institute of Orthopedics “Carlos E. Ottolenghi,” Italian Hospital of Buenos Aires, Juan D. Peron 4190, C1181ACH Buenos Aires, Argentina

In the article titled “Migration of the Anterior Spinal Rod to the Right Thigh, a Rare Complication of Anterior Spinal Instrumentations: A Case Report and a Literature Review” [1], the authors' first and last names were reversed. The corrected authors' list is shown above.
